# From Waste to Taste: Application of Fermented Spent Rootlet Ingredients in a Bread System

**DOI:** 10.3390/foods12071549

**Published:** 2023-04-06

**Authors:** Emma Neylon, Laura Nyhan, Emanuele Zannini, Aylin W. Sahin, Elke K. Arendt

**Affiliations:** 1 School of Food and Nutritional Science, University College Cork, T12K8AF Cork, Ireland; 2 Department of Environmental Biology, “Sapienza” University of Rome, 00185 Rome, Italy; 3 APC Microbiome Ireland, University College Cork, Western Road, T12K8AF Cork, Ireland

**Keywords:** brewing by-products, malting by-products, by-product valorisation, lactic acid bacteria fermentation, circular bio-economy, sustainability

## Abstract

The process of upcycling and incorporating food by-products into food systems as functional ingredients has become a central focus of research. Barley rootlets (BR) are a by-product of the malting and brewing industries that can be valorised using lactic acid bacteria fermentation. This research investigates the effects of the inclusion of unfermented (BR-UnF), heat-sterilised (BR-Ster), and five fermented BR ingredients (using *Weissella cibaria* MG1 (BR-MG1), *Leuconostoc citreum* TR116 (BR-TR116), *Lactiplantibacillus plantarum* FST1.7 (BR-FST1.7), *Lactobacillus amylovorus* FST2.11 (BR-FST2.11), and *Limosilactobacillus reuteri* R29 (BR-R29) in bread. The antifungal compounds in BR ingredients and the impact of BR on dough rheology, gluten development, and dough mixing properties were analysed. Additionally, their effects on the techno-functional characteristics, in vitro starch digestibility, and sensory quality of bread were determined. BR-UnF showed dough viscoelastic properties and bread quality comparable to the baker’s flour (BF). BR-MG1 inclusion ameliorated bread specific volume and reduced crumb hardness. Breads containing BR-TR116 had comparable bread quality to BF, while the inclusion of BR-R29 substantially slowed microbial spoilage. Formulations containing BR-FST2.11 and BR-FST1.7 significantly reduced the amounts of sugar released from breads during a simulated digestion and resulted in a sourdough-like flavour profile. This study highlights how BR fermentation can be tailored to achieve desired bread characteristics.

## 1. Introduction

In recent years, by-product rejuvenation has become a pivotal point of food research to align with the sustainability goals of the future. Spent barley rootlets (BR) are the main by-products produced during the malting process and can be generated in volumes that equate to up to 5% of the total malt weight [1,2]. Due to the fundamental role of the malting process within the brewing industry, BR are also classified as a brewing by-product. To date, the use of BR has been rather limited, with its primary output confined to the animal feed industry. However, BR are of excellent nutritional value, containing a high amount of fibre (9.70–43%) [3] and substantial amounts of protein (20–38.7%) [4], highlighting their potential as food ingredients for inclusion in the human diet.

Although limited studies exist in the literature regarding the application of BR in food matrices, the implementation of BR into the food chain has promising potential from a nutritional perspective, which can increase the protein and fibre values of foods [5]. However, the inclusion of BR in food products poses difficulties, with losses in food quality and adverse sensory perceptions observed. Chiş et al. [6] found the inclusion of BR up to 15% was successful, as undesirable sensory defects were noted beyond this point. Salama et al. [5] reported maximum inclusion levels of BR in biscuits (10%), bread (5%) and sausages (10%), with additional levels beyond these resulting in products being deemed unacceptable by sensory panellists. Similarly, Waters et al. [3] found that a 5% addition of BR was the most satisfactory from a sensory perspective, as anything above this level exhibited bitter off-flavours. From a bread quality perspective, the introduction of BR has resulted in lower bread volumes, harder crumb textures, and darker crust colours [3].

Lactic acid bacteria (LAB) fermentation has become a process of interest in the enhancement of side-stream products, with numerous studies showcasing their successes [7,8,9,10,11,12,13,14,15,16,17,18]. The application of LAB fermentation technology to brewers’ spent grain and apple by-products showed improvements in a variety of nutritional characteristics, enhanced bread and pasta techno-functional properties, extended shelf-life properties, and also produced acceptable sensory attributes [7,8,9,10,11,12,13,14]. LAB fermentation of surplus bread streams and their incorporation into a bread matrix showed improvements in bread specific volume, reduced crumb hardness, and also improved the hygienic safety of the recycling process [15]. The application of LAB fermentation technology to side streams from the maize and wheat milling industries and their incorporation into wheat and pasta prototypes exhibited improvements in product quality, nutritional properties, and sensory attributes compared to their unfermented control [16,17,18]. About BR, the application of LAB sourdough fermented BR at 10% in a bread matrix displayed enhanced bread texture and flavour quality compared to wholemeal bread [3].

Neylon et al. [19] performed an in-depth analysis of the effect of heat treatment and LAB fermentation (using *Weissella cibaria* MG1, *Lactiplantibacillus plantarum* FST1.7, *Limosilactobacillus reuteri* R29, *Leuconostoc citreum* TR116, and *Lactobacillus amylovorus* FST2.11) on BR. Following this, the current study aimed to investigate the effects of the inclusion of fermented BR ingredients in a bread matrix. Fermented BR ingredients were incorporated into bread recipes at a 5% replacement level based on bakers’ wheat flour. The effect of BR addition on dough quality, techno-functional characteristics, microbial shelf-life, sensory experience, and nutritional characteristics (release of reducing sugars during starch digestion) of bread were monitored. Formulations containing bakers’ wheat flour (BF), unfermented BR (BR-UnF), and heat-sterilised BR (BR-Ster) were used as controls.

## 2. Materials and Methods

### 2.1. Raw Materials

Ingredients incorporated into bread recipes included bakers’ flour (BF) (Odlums Group, Dublin, Ireland); spent barley rootlets (UnF-BR) supplied by Anheuser-Busch InBev (Leuven, Belgium); a sterilised, freeze-dried BR ingredient (BR-Ster); and five fermented, freeze-dried BR ingredients [19]. The fermented, freeze-dried BR ingredients were produced using LAB fermentation with the strains listed in Table 1, as described by Neylon et al. [19]. Briefly, 12.5% (*w/v*) of BR-UnF were added to water to a final volume of 800 mL and transferred to a 1 L batch bioreactor on a DASGIP Bioblock equipped with DASGIP TC4SC4 and DASGIP PH8 modules (Eppendorf, Stevenage, UK). A total of 40 g (5% *w/v*) of the appropriate sugar source was incorporated in the rootlet/water mixture. A sterilisation process of the rootlet/water/sugar mixture was performed at 90 °C for 30 min, cooled to 30 °C, and the selected LAB strain was inoculated (final cell concentration: 10^7^ CFU/mL). The BR fermentation was performed at 30 °C for 96 h using a continuous mixing (400 rpm) process. After completion of fermentation, BR mixtures were pasteurised (72 °C, 15 min) and finally freeze-dried. Along with BR, bread recipes also included instantaneous freeze-dried yeast (Puratos, Groot-Bijgaarden, Dilbeek, Belgium); granulated sugar (Siúcra, Dublin, Ireland); salt (Glacia British Salt Limited, Cheshire, UK); sunflower oil (Musgraves, Cork, Ireland); and regular faucet water. The compositional analyses of the BF and BR-UnF are illustrated in Table 2, with the composition of the BR-UnF previously reported by Neylon et al. [19]. The compositional analysis was carried out externally by a certified lab (Chelab S.r.l., Merieux NutriSciences Corporation, Resana TV, Italy). For the analysis of proteins in BR, the Dumas method was used, adapting an amended version of AOAC 992.23 (1992) [20]. The level of fat present in BR was determined with Soxhlet and was conducted in line with the ISTISAN report [21]. Ash quantities were assessed with AOAC 923.03 [22]. The level of moisture in BR was calculated using ISO 712:2009 [23]. The sum of carbohydrates was calculated using a difference-based method (AOAC 986.25) [24]. The total fibre content was measured by the AOAC 2017.16 [25]. Except where specified, Sigma-Aldrich (Sigma-Aldrich, St.Louis, MO, USA) supplied chemicals utilised for experiments.

### 2.2. Analysis of Dough Properties

#### 2.2.1. Water Content

The Farinograph-TS^®^ (Brabender GmbH and Co. KG, Duisburg, Germany) was utilised to adjust the amount of water required for each recipe using an automatic water dosing system (Aqua-Inject. Brabender GmbH and Co. KG, Duisburg, Germany). Recipes for a total volume of 300 g were adjusted to a target dough consistency of 500 ± 20 FU with the mixing chamber temperature set to 30 °C. A 5% addition (based on flour) of the respective BR ingredient was included in bread recipes by replacing BF. The bread recipes used for analysis are included in Table 3.

#### 2.2.2. Preparation of Dough

Bread doughs were produced by a straight dough process [47]. Firstly, the dry ingredients were mixed using a Kenwood Chef Classic mixer (Kenwood Manufacturing Co., Ltd., Havant, UK) for 30 s at speed 1. Following this, the yeast solution and sunflower oil were added to the dry ingredients. The solution of yeast was made by adding the dry yeast to the amount of water required for the recipe (tempered to 25 °C) and allowing it to stand for 10 min to activate the yeast. All ingredients were mixed at speed 1 for 1 min, and directly after, a second mixing stage at speed 2 for 7 min was carried out.

#### 2.2.3. Dough Rheology

The viscoelastic properties of the doughs were evaluated using a Rheometer Physica MCR 301 (Anton PAAR GmBH, Ostfildern, Germany). Bread doughs were prepared as outlined above, but with the yeast excluded. A serrated plate method was employed for the analysis, with the plates set up in parallel geometry. The lower plate was held at 35 °C and was assembled with an upper plate of 50 mm in diameter. The linear viscoelastic region was evaluated using an amplitude sweep [48], and frequency sweeps were carried out as described by Neylon et al. [9]. The damping factor ($tan\delta \frac{G^{\prime \prime }}{G^{\prime }}$) was determined to analyse the changes in dough viscoelastic properties caused by BR ingredient addition.

#### 2.2.4. Bread Fermentation Quality

The bread dough quality was analysed using a Rheofermentometer (Chopin, Villeneuve-la-Garenne CEDEX, France). Briefly, 300 g of bread dough was prepared, positioned in the fermentation chamber, and a 1500 g cylindrical weight was placed on top of the dough. The fermentation chamber was sealed, and the BR doughs were left to ferment for 3 h at 30 °C. The maximum dough height (Hm) in mm, CO_2_ retention coefficient (%), and CO_2_ volume generated during fermentation (mL) were analysed.

#### 2.2.5. Dough Development and Starch Pasting Properties

The mixing and pasting behaviour of the doughs were analysed using Mixolab (Chopin, Villeneuve-la-Garenne CEDEX, France) according to the method detailed by Rosell et al. [49]. Briefly, flour blends (Table 3) were mixed with the required water content according to Farinograph-TS^®^ values (Table 3) to reach a dough weight of 75 g. Samples were mixed at a constant speed of 75 rpm. A heating profile was applied to the dough starting at 30 °C until maximum dough development, heated to 90 °C over 15 min at a rate of 4 °C/min, and held at 90 °C for a total of 7 min. A cooling profile was then applied over 10 min to 50 °C at a rate of 4 °C/min and held at 50 °C for a total of 5 min. The following parameters were analysed: dough development time (DDT), C2, C3, C4, and C5. As described by Rosell et al. [50], DDT is determined as the time taken (min) for the maximum torque to be reached during the first stage of mixing at a temperature of 30 °C. C2 is the minimum value of torque when the dough is exposed to mechanical and temperature stress and provides information on protein destabilisation or protein weakening. C3 is the maximum torque reached during the heating and mixing stage, which provides information on the starch swelling and hydration properties. C4 is deemed the minimum torque reached and provides an insight into the physical breakdown of the starch granules in the dough. Finally, C5 is the final maximum torque reached during the cooling phase, providing information on the extent of starch retrogradation.

#### 2.2.6. Gluten Network Development

The gluten network aggregation was evaluated using GlutoPeak (Brabender GmbH and Co., KG, Duisburg, Germany). The flour blends (Table 3) were prepared and mixed to ensure homogeneity. For analysis, 9 g of the sample (based on 14% moisture) was added to deionized water (36 °C) to reach a final volume of 18 g. Measurement parameters were set to a shear speed of 2750 rpm at a chamber temperature of 36 °C. Torque was analysed over time (s) with torque maximum (TM) in Brabender units (BU) and peak maximum time (PMT) in seconds (s) evaluated.

### 2.3. Bread Production Process

For each recipe outlined in Table 3, dough quantities of 550 g were prepared. The bread dough was divided into eight 65 ± 1 g pieces, moulded, and placed into greased tins (90 × 50 × 40 mm). The dough pieces were proofed (30 °C, 85% relative humidity (RH)) in a proofing chamber (KOMA SunRiser, Roermond, The Netherlands) for 75 min. After proofing, the doughs were transferred to a deck oven (MIWE Condo, Arnstein, Germany) and baked (210 °C top/bottom heat, 14 min). To optimise conditions during baking, 700 mL of steam was loaded into the oven before the breads were placed in it. After baking, the bread loaves were allowed to cool for 1 h before analysis.

### 2.4. Bread Analysis

#### 2.4.1. Bake Loss

Bake loss was calculated to account for the amount of water lost during the baking process. The bake loss of four loaves per batch was analysed and calculated according to the below formulas:

Weight of the dough (g)−weight of baked bread (g)=moisture lost during bake (g)

$$\frac{Moisture\;lost\;during\;bake\;\left(g\right)}{Weight\;of\;dough\;\left(g\right)}\times 100=Bake\;loss\;\left(\%\right)$$

#### 2.4.2. Specific Volume

The specific volume of each bread loaf was determined using the Volscan Profiler (Stable Micro Systems, Surrey, UK) and expressed as mL/g. Four bread loaves were analysed per batch.

#### 2.4.3. Breadcrumb Structure

The C-Cell Imaging System (Calibre Control International Ltd., Warrington, UK) was used to analyse the cell diameter of the breads. Four bread loaves per batch were cut into slices of 20 mm thickness (three slices per loaf), with crust slices omitted from the analysis.

#### 2.4.4. Breadcrumb Texture

The crumb texture of bread slices was measured using the TA-XT2i Texture Analyser (Stable MicroSystems, Surrey, UK). The TA-XT2i Texture Analyser, together with a 25 kg load cell, conducted a two-compression test with a strain of 40%, a test speed of 5 mm/s, and a trigger force of 0.05 N. A waiting time of 5 s between the two compressions was used. A 20 mm cylindrical probe was attached for the analysis. Bread slices of 20 mm thickness were analysed for their hardness and resilience after the breads had cooled. The crumb hardness was determined as the maximum force of the first compression. Breadcrumb resilience was determined by dividing the energy required during the upstroke action of compression one by the energy required during the downstroke action of compression one.

### 2.5. Extraction and Quantification of Antifungal Compounds from BR Ingredients

A total of 15 known antifungal phenolic compounds were tested for their presence in BR ingredients [51], including 4-hydroxybenzoic acid, hydrocaffeic acid, 4-hydroxyphenyllactic acid, catechol, phloretic acid, vanillic acid, 3-phenyllactic acid, caffeic acid, hydroferulic acid, benzoic acid, hydrocinnamic acid, p-coumaric acid, ferulic acid, salicylic acid, and methylcinnamic acid. The QuEChERS (quick, easy, cheap, effective, rugged, and safe) method was adopted [51], according to the modified version outlined by Hoehnel et al. [26]. Briefly, 2 g of BR was mixed in 10 mL of ultrapure H_2_O along with 10 mL of ethyl acetate containing 0.1% (*v*/*v*) formic acid. The samples were mixed thoroughly using a vortex shaker. Following this, 4 g of MgSO_4_ and 1g of NaCl were added, and the samples were shaken by hand for exactly 1 min. Afterwards, samples were centrifuged (4800× *g*, 10 min). The supernatant was placed in solid-phase extraction tubes (Bond Elut QuEChERS Dispersive kit; Agilent Technologies Inc., Santa Clara, CA, USA). A vortex shaker was used to distribute the contents of the supernatant in the tubes, which were then centrifuged (2300× *g*, 10 min). Directly after, a total of 5 mL of supernatant was combined with 100 µL of dimethlsulfoxide. In a vacuum centrifuge (Scanvac Scanspeed, Labogene, Lillerød, Denmark), solvents present in samples were allowed to evaporate for two hours at 500 rpm and 45 °C before reconstitution with 400 µL of ultrapure H_2_O/acetonitrile (90:10, *v*/*v*). A syringe-driven filter (0.2 µm) was used to clarify the samples. Evaluation of the antifungal compound profiles of BR was performed according to chromatographic test parameters detailed by Hoehnel et al. [26]. Antifungal quantities were calculated based on dry matter (D.M.).

### 2.6. Shelf-Life Evaluation

The microbial shelf-life of the breads was measured using the mould environmental challenge method [41,44,52] with some minor modifications. Briefly, eight centre slices of 20 mm thickness per batch were left to rest on a sterilised stain-less steel rack. Breadcrumbs from both sides of the bread slice were exposed to the bakery air for 5 min. The bread slices were packed singly in sterile bags and heat-sealed, and to facilitate airflow for aerobic conditions, a filter pipette was sterilely inserted in all bags. The samples were stored at 20 ± 1 °C and 50% RH in a pre-sterilised and temperature-controlled chamber (KOMA SunRiser, Roermond, The Netherlands). Samples were analysed daily for 14 days. The mould growth progression of all bread slices was visually assessed and rated as “mould free”, “mould growth < 10%”, “10–24% mould growth”, “25–49% mould growth” and “mould growth > 50%”.

### 2.7. Release of Reducing Sugars

The release of reducing sugars was investigated by means of an in vitro digestion assay using an enzymatic degradation of starch. The method was carried out as previously described by Brennan & Tudorica [53], which was designed for fibre-enriched products. Briefly, 4 g of ground breadcrumbs were treated with a pepsin solution (115 U/mL) for 30 min at 37 °C. Following this, samples were placed in dialysis tubing (1-inch diameter, molecular weight cut off at 14,000 Daltons) and suspended in a sodium potassium phosphate buffer (pH 6.9) and incubated with pancreatic alpha-amylase solution (110 U/mL). During the incubation period, the dialysis tubing was inverted three times to simulate the peristalsis effect. Samples were collected from the buffer solution at 30 min intervals. A 100 µL aliquot of sample was diluted with a 100 µL sample of 3,5-dinitrosalicyclic acid and heated (100 °C, 15 min). Following this, the heated samples were left to cool for 5 min on ice and were subsequently diluted with 1 mL of deionised H_2_O. The absorbance of the samples at 546 nm was measured, and the reducing sugar release % (RSR) over time was determined using the below formula [53].

$$RSR\;\%=\frac{(Asample)\;\times \;500\;\mathrm{mL}\;\times \;0.95}{\left(A\;maltose\right)\;\times \;available\;carbohydrate\left(\mathrm{mg}\right)\;}\times 100$$

where (A sample) is the absorbance of the active sample at 546 nm, 500 mL is the total volume of the solution that was analysed, 0.95 is the conversion factor for maltose to starch, (A maltose) is the absorbance of 1 mg pure maltose/mL buffer, and available carbohydrates is the amount of readily digestible carbohydrates present in the 4 g sample. The available carbohydrate was determined using the digestible carbohydrate values obtained from the K-RAPRS kit (Megazyme, Bray, Ireland).

### 2.8. Sensory Evaluation

The sensory properties of the breadcrumbs were investigated by performing a descriptive analysis test using an experienced sensory panel (*n* = 9, age range 23–33) from the Food Science department of the University College Cork. Panellists evaluated the intensity of the odour attributes ‘overall intensity, ‘citrus’, ‘vegetables, ‘cereals/grains’, the flavour attributes ‘overall intensity’, ‘muddy/earthy’, ‘fruity’, ‘vegetables’, ‘aftertaste’, the taste attribute ‘sour’, and the texture attributes ‘hardness’, ‘chewiness’ on a scale from 0, ‘not present’ to 10, ‘extremely present’. Panellists were also asked to rank the overall acceptability of the breads. Sensory analysis of all breads was performed in duplicate, and breadcrumb samples were analysed on the same day they were baked. The questionnaire used during sensory analysis is available in the Supplementary Materials (Figure S1).

### 2.9. Statistical Analysis

Experimental analysis was carried out in triplicate unless stated otherwise. In the case of normally distributed data, a one-way ANOVA and post-hoc Tukey test (*p* value < 0.05) were completed using SPSS statistical software (version 28.0.1.0, IBM SPSS, Chicago, IL, USA) in order to determine significant differences between sample groups. A Welch test correction and Games Howell post hoc test (*p* < 0.05) were performed on instances where equal variances could not be assumed. A Kruskal-Wallis test (*p* < 0.05) was utilised when data did not conform to normal distribution.

## 3. Results

### 3.1. Compositional Analysis

The compositions of BF and BR-UnF are presented in Table 2. The compositional analysis of BR-UnF has been reported in a previous publication [19]. The protein content in BR-UnF (35.80 ± 1.50 g/100 g) was much higher than the protein content quantified in BF (12.96 ± 0.79 g/100 g). BF and BR-UnF had relatively similar quantities of fat (1.20 ± 0.08 g/100 g and 1.77 ± 0.11 g/100 g, respectively); however, higher amounts of ash were quantified in BR-UnF (5.98 ± 0.30 g/100 g) compared to BF (0.55 ± 0.05 g/100 g). The moisture contents measured in BF (12.95 ± 0.30 g/100 g) and BR-UnF (12.74 ± 0.30 g/100 g) were comparable. BF contained 65.31 ± 1.53 g/100 g of total carbohydrate, which was much lower than that measured in BR-UnF (0 ≤ 7.02 ≤ 15.72 g/100 g). The total fibre in BF equated to 7.03 ± 1.27 g/100 g, of which 2.63 ± 0.63 g/100 g was deemed as soluble fibre and 4.41 ± 1.1 g/100 g was determined as high molecular weight dietary fibre. Higher quantities of total fibre (36.64 ± 8.51 g/100 g) and high molecular weight dietary fibre (35.40 ± 8.50 g/100 g) were quantified in BR-UnF, with a slightly lower amount of soluble fibre quantified in BR-UnF (1.24 ± 0.30 g/100 g) than BF.

### 3.2. Dough Analysis

#### 3.2.1. Water Absorption

The optimal water content for each recipe was determined to ensure optimal dough consistency for favourable dough and gluten network development. The results from the farinograph analysis are displayed in Table 3. The lowest water absorption capacity (WAC) was found in the BF recipe (64.3%). The inclusion of the BR ingredients significantly increased the WAC of the recipes. The highest WAC was determined in the BR-Ster recipe (68.9%), followed closely by the BR-UnF recipe (68.2%), with no significant differences found between these samples. The WAC of the BR-UnF recipe and the recipes containing BR-MG1 (67.90%), BR-FST2.11 (67.50%), and BR-FST1.7 (67.30%) were similar. A WAC of 66.90% and 66.70% was measured in the BR-TR116 and BR-R29 recipes, respectively, which were significantly lower than the BR control recipes, BR-UnF and BR-Ster.

#### 3.2.2. Gluten Network Development

**Figure 1:**
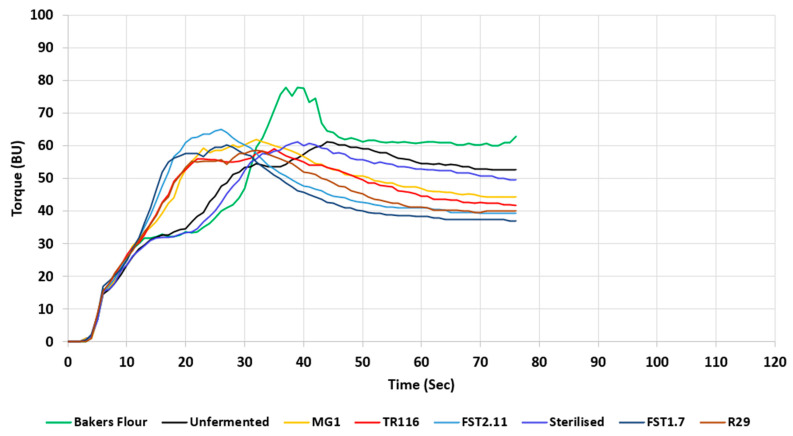
Effect of unfermented and fermented rootlets on the gluten network development of bakers’ flour as dough torque (BU) over mixing time (s). TR116, FST2.11, MG1, FST1.7, and R29 denote wheat flour blends with 5% inclusion of the fermented rootlets, fermented with *Leuconostoc citreum* TR116, *Lactobacillus amylovorus* FST2.11, *Weissella cibaria* MG1, *Lactiplantibacillus plantarum* FST1.7, and *Limosilactobacillus reuteri* R29, respectively. Bakers’ flour, 5% supplementation of unfermented rootlets, and 5% supplementation of sterile rootlet flour blends are also represented in the illustration.

Attributes of the gluten network’s development were determined to investigate any differences in gluten aggregation kinetics. The torque maximum (TM) and the time taken for gluten development (PMT) are shown in Table 4, while the torque (BU) versus time (s) of the gluten network development is displayed in Figure 1. A curve typical for wheat flour was obtained for BF with a TM of 78.33 ± 1.54 BU and a PMT of 39.67 ± 1.53 s. The inclusion of BR-UnF and BR-Ster significantly reduced the gluten network strength, with TM values of 64.00 ± 1.00 BU and 61.67 ± 2.53 BU, respectively. No significant differences were noted in the times required for gluten development with BF (39.67 ± 1.53 s), BR-UnF (44.00 ± 1.00 s), and BR-Ster (40.00 ± 2.00 s). The inclusion of fermented BR ingredients resulted in decreased gluten network strength compared to the BF control formulation but was similar to the BR-UnF and BR-Ster results. The weakest gluten aggregation strength was noted in the BR-R29 (58.67 ± 1.54 BU) recipe, followed closely by recipes with BR-FST1.7 (60.00 ± 1.00 BU) and BR-TR116 (60.33 ± 4.04 BU) inclusion. An increase in gluten strength was found for BR-MG1 formulations (62.33 ± 1.53 BU), with the strongest gluten network amongst the fermented formulations observed with BR-FST2.11 (65.33 ± 1.16 BU) inclusion. Concerning the gluten network development time, all fermented ingredients enhanced the speed of gluten aggregation in the dough, resulting in a decrease in PMT compared to BF, BR-UnF, and BR-Ster. The fastest gluten network development was observed in formulations supplemented with BR-FST2.11 (25.67 ± 1.16 s) and BR-FST1.7 (26.67 ± 1.16 s), with no significant differences found. The PMT observed for BR-TR116 (33.67 ± 3.215 s), BR-MG1 (32.67 ± 2.08 s), and BR-R29 (32.33 ± 0.58 s) recipes were significantly longer than those of fermented samples BR-FST1.7 (26.67 ± 1.16 s), and BR-FST2.11 (25.67 ± 1.16 s). However, they were still significantly faster than the PMT values obtained for the BF, BR-UnF, and BR-Ster controls.

#### 3.2.3. Dough Development and Starch Pasting Properties

The Mixolab analyses were conducted to investigate the effects observed in dough mixing characteristics and pasting properties under mechanical and thermal stresses (Table 4).

The shortest DDT was observed for the BF formulation (1.32 ± 0.27 min), with significantly higher DDT observed in the BR-UnF (7.04 ± 0.23 min) and BR-Ster (7.49 ± 0.32 min) formulations. Significant reductions in DDT (3.66–5.61 min) were determined with the inclusion of most fermented BR ingredients. The BR-MG1 formulation was the exception to this trend, with no significant difference in DDT noted when compared to the BF, BR-UnF, and BR-Ster formulations.

The BF control recipe resulted in the highest C2 value (0.427 ± 0.01 Nm), with a significant reduction in C2 values recorded with the inclusion of both BR-UnF (0.33 ± 0.00 Nm) and BR-Ster (0.38 ± 0.03 Nm). Comparing fermented formulations, the C2 values for doughs containing BR-MG1 (0.36 ± 0.00 Nm) and BR-TR116 (0.35 ± 0.01 Nm) were the highest. C2 values for BR-MG1 and BR-TR116 recipes were significantly lower than BF and BR-Ster, however, but were significantly higher than the BR-UnF formulation. The inclusion of BR-R29 reduced the C2 value to 0.34 ± 0.00 Nm, with an even greater reduction observed with doughs containing BR-FST2.11 (0.31 ± 0.01 Nm) and BR-FST1.7 (0.30 ± 0.01 Nm).

BF had the highest C3 value recorded (1.63 ± 0.01 Nm), which was significantly higher than the BR-Ster value (1.52 ± 0.01 Nm). In contrast, the BR-UnF formulation had the lowest C3 value (1.24 ± 0.02 Nm). C3 values for fermented formulations were in the range of 1.53–1.63 Nm.

Comparing C4 values, BR-UnF had the lowest value recorded (0.71 ± 0.03 Nm). A significant increase in C4 was observed for the BR-Ster formulation (1.38 ± 0.01 Nm), with an even greater increase reported for BF (1.50 ± 0.01 Nm). C4 values for BR-MG1 (1.41 ± 0.01 Nm) were comparable to the BR-Ster formulation. No significant difference was found between BR-MG1 and BR-TR116 (1.44 ± 0.01 Nm). The BR-R29 (1.47 ± 0.01 Nm), BR-FST1.7 (1.50 ± 0.01 Nm), and BR-FST2.11 (1.51 ± 0.01 Nm) C4 values were comparable to the BF recipe.

Trends for C5 values mirrored those reported for C4 values. BR-UnF doughs had the lowest C5 value recorded (1.12 ± 0.03 Nm), while significant increases were observed in C5 values for doughs containing BR-Ster (2.23 ± 0.03 Nm) and BF (2.55 ± 0.03 Nm). C5 values for fermented formulations were in the range of 2.28–2.65 Nm.

#### 3.2.4. Bread Fermentation Capacity

The fermentation capacity of each dough was analysed to provide information on the yeast fermentation process (Table 4).

Of the formulations tested, BF had the highest Hm (50.93 ± 2.48 mm). The inclusion of BR-UnF maintained the dough height (46.40 ± 1.31 mm), with no significant difference observed compared to the BF control. A greater reduction in Hm was observed when BR-Ster was included (40.43 ± 0.57 mm). Comparing fermented BR formulations, the highest Hm was recorded with BR-MG1 inclusion (44.87 ± 1.52 mm) and was comparable to all control recipes. The inclusion of BR-TR116 resulted in a slight decrease of Hm (43.17 ± 3.26 mm), but was not significantly different from the BR-MG1 Hm results. Compared to BF and BR-UnF, the addition of BR-FST2.11, BR-R29, and BR-FST1.7 reduced Hm significantly, with values of 37.00 ± 0.30 mm, 36.57 ± 2.53 mm, and 34.97 ± 3.10 mm, respectively. However, these values were not found to be statistically different from the Hm of the BR-Ster recipe. The volume of CO_2_ recorded varied between recipes. Firstly, comparing controls, the lowest amount of CO_2_ produced was recorded in the BF recipe (2045 ± 73 mL), while significant increases were observed in the volume of CO_2_ produced with BR-UnF (2451 ± 30 mL) and BR-Ster (2399 ± 100 mL) inclusion. The incorporation of BR-MG1 produced the highest amount of CO_2_ during the analysis (2547 ± 68 mL) and was comparable to the BR-UnF and BR-Ster controls. Similarly, the BR-FST2.11 and BR-FST1.7 formulations maintained high amounts of CO_2_ production, resulting in values of 2318 ± 131 mL and 2302 ± 172 mL, respectively. A slight drop in the level of CO_2_ produced was observed in the BR-TR116 recipe (2029 ± 69 mL), which was more comparable to the BF and BR-FST1.7 formulations. The lowest amount of CO_2_ produced was noted in the BR-R29 recipe (1919 ± 59 mL), which was similar to the volume of CO_2_ produced in the BF fermentation process. Finally, the CO_2_ retention coefficient for all recipes was comparable, with no significant difference found between them.

#### 3.2.5. Dough Rheology

To investigate the dough’s elastic (solid) and viscous (liquid) parts, oscillation measurements were conducted, and the damping factor (DF) was determined. A material is considered ideal and elastic if the damping factor is 0, implying that the material contains no viscous elements. An increase in the DF signifies an increase in the viscous parts of the dough system.

As shown in Table 4, BF had the highest DF (0.390 ± 0.012), indicating the highest viscosity in this dough. The BR-UnF formulation showed similar dough rheological properties (0.383 ± 0.009) to BF. The DF determined in the BR-Ster formulation was significantly lower (0.357 ± 0.010) than the BF and BR-UnF formulations, indicating a dough with more elastic properties. The inclusion of fermented BR ingredients resulted in a significant reduction in the DF compared to the BF and BR-UnF formulations. However, no significant difference was noted in the BR-TR116 (0.346 ± 0.012), BR-MG1 (0.352 ± 0.011), and BR-R29 (0.344 ± 0.011) formulations compared to the BR-Ster control. A significant reduction in DF was found in BR-FST1.7 (0.333 ± 0.014) and BR-FST2.11 (0.328 ± 0.011) formulations compared to control recipes, with the lowest DF noted in the BR-FST2.11 formulation.

### 3.3. Baked Bread Analysis

**Figure 2:**
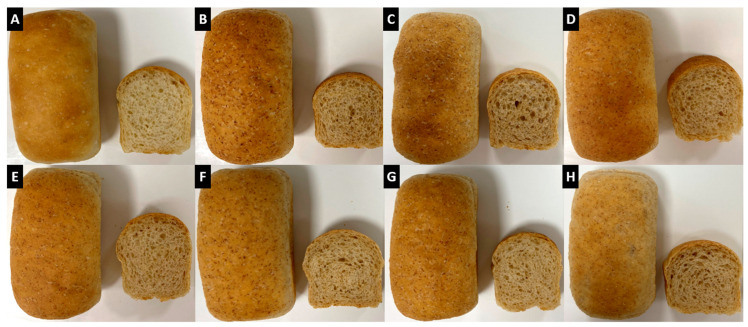
Images of rootlet breads produced during baking trials. Images (**A**–**C**) represent control recipe breads made with bakers’ flour, unfermented barley rootlets, and sterilised barley rootlets, respectively. Images (**D**–**H**) illustrate breads made with fermented barley rootlets with the strains *Leuconostoc citreum* TR116, *Weissella cibaria* MG1, *Lactobacillus amylovorus* FST2.11, *Limosilactobacillus reuteri* R29, and *Lactiplantibacillus plantarum* FST1.7, respectively.

A visual representation of the final baked bread products is illustrated in Figure 2.

#### 3.3.1. Baking Loss

Bake loss (BL) was determined to analyse the extent of water loss during the bake. Results from this analysis are depicted in Table 5. BF bread had a BL of 14.24 ± 0.91%, which was not significantly different from any of the formulations tested. A similar BL was observed in BR-UnF (13.54 ± 0.58%) and BR-Ster (13.56 ± 0.53%), with minor differences noted between samples. Among fermented BR ingredient formulations, the highest BL was observed in the BR-TR116 formulation (14.33 ± 0.47%), followed closely by BR-MG1 (14.19 ± 0.38%) and BR-FST2.11 (13.85 ± 0.31%). A minor reduction in BL was observed in BR-R29 (13.36 ± 0.58%) and BR-FST1.7 (13.54%) formulations; however, all were comparable to the BF control.

#### 3.3.2. Specific Volume

The specific volume of each bread was analysed as an important parameter to investigate the rise and expansion of the bread. The results from the specific volume analysis are presented in Table 5. Comparing control bread, BF and BR-UnF formulations had similar specific volumes, with values of 3.74 ± 0.20 mL/g and 3.55 ± 0.14 mL/g measured, respectively. A significant reduction in the specific volume was noted with the inclusion of BR-Ster (3.09 ± 0.18 mL/g).

Concerning the fermented ingredients, the formulation including BR-MG1 resulted in the highest specific volume recorded (3.80 ± 0.13 mL/g), which was comparable to BF and significantly higher than the BR-UnF and BR-Ster controls. BR-TR116 had a specific volume of 3.68 ± 0.22 mL/g, which was comparable to BF and BR-UnF. A significant reduction in specific volume was observed in BR-R29 (3.35 ± 0.29 mL/g). The lowest specific volumes recorded were in BR-FST2.11 (2.80 ± 0.15 mL/g) and BR-FST1.7 (2.63 ± 0.19 mL/g) formulations, which were significantly lower than BF, BR-UnF, and BR-Ster control recipes.

#### 3.3.3. Crumb Structure-Cell Diameter

The cell diameter was determined in order to provide information on the cell structure of the crumb. Results are depicted in Table 5.

The largest cell diameter was determined in the BF control bread (2.93 ± 0.15 mm). A reduction in cell diameter was observed in BR-UnF (2.16 ± 0.15 mm) and BR-Ster (1.94 ± 0.18 mm) bread compared to the BF.

Amongst the fermented formulations, BR-MG1 had the highest cell diameter (2.05 ± 0.16 mm), which was not significantly different from the BR-UnF and BR-Ster controls. The BR-TR116 formulation had a cell diameter of 1.95 ± 0.17 mm, followed by BR-FST2.11 (1.83 ± 0.22 mm), BR-R29 (1.83 ± 0.15 mm), and BR-FST1.7 (1.63 ± 0.16 mm).

#### 3.3.4. Breadcrumb Texture

The breadcrumb texture was determined to provide information on the breadcrumb quality. Results of crumb texture parameters hardness and resilience are represented in Table 5.

##### Hardness

Among the control recipes, the softest crumb was noted in the BR-UnF formulation (1.77 ± 0.26 N). A significant increase in breadcrumb hardness was observed in BF (2.33 ± 0.32 N), with an even larger increase recorded in the BR-Ster (3.14 ± 0.42 N).

The lowest breadcrumb hardness was found in BR-MG1 (1.61 ± 0.22 N), followed by BR-TR116 (1.96 ± 0.30 N), with both recipes having comparable crumb hardness to the BR-UnF formulation. An increase in crumb hardness was found in the BR-R29 formulation (2.39 ± 0.35 N), which was more comparable with the BF control recipe. In the BR-FST2.11 and BR-FST1.7 recipes, a significant increase in crumb hardness was observed, with hardness values of 3.59 ± 0.53 N and 4.11 ± 0.55 N recorded, which were aligned with the BR-Ster hardness value.

##### Resilience

The highest breadcrumb resilience was noted in the BF control (0.56 ± 0.01 N), with some reductions in breadcrumb strength found in BR-UnF (0.53 ± 0.02 N) and BR-Ster (0.55 ± 0.01 N). Comparing fermented ingredients, the inclusion of BR-MG1 resulted in the highest breadcrumb resilience (0.56 ± 0.01 N). A reduction in breadcrumb strength was observed with BR-TR116 (0.55 ± 0.02 N) and BR-R29 inclusion (0.54 ± 0.01 N), aligning with BR-Ster and BR-UnF values. BR-FST2.11 (0.52 ± 0.02 N) and BR-FST1.7 (0.53 ± 0.01 N) inclusion had the greatest reduction in crumb strength, which was significantly different from BF, BR-UnF, and BR-Ster formulations.

#### 3.3.5. Microbial Shelf-Life Properties

##### Antifungal Compounds in BR Ingredients

The BR ingredients used in the study were screened for the presence of 15 known phenolic-type antifungal compounds to investigate naturally present antifungal compounds as well as the extent of those produced during LAB fermentation. Results from the analysis are presented in Table 6.

Hydroxyphenyllactic acid was not detected in quantifiable amounts in BR-UnF, BR-Ster, or BR-MG1. The highest amount of hydroxyphenyllactic acid was determined in BR-FST1.7 (9.136 ± 0.104 g/100 g D.M.). In comparison to BR-FST1.7, a significant reduction in hydroxyphenyllactic was observed in BR-FST2.11 (2.483 ± 0.247 g/100 g D.M.) and BR-R29 (2.239 ± 0.087 g/100 g D.M.), with the lowest amount recorded in BR-TR116 (0.637 ± 0.091 g/100 g D.M.). Concerning 4-hydroxybenzoic acid, the highest amount detected was in BR-Ster (1.215 ± 0.190 g/100 g D.M.). No significant differences were found in levels of 4-hydroxybenzoic acid between BR-Ster and BR-UnF (0.928 ± 0.006 g/100 g D.M.). Among the fermented ingredients, relatively similar amounts of 4-hydroxybenzoic acid were determined. BR-TR116 (1.177 ± 0.036 g/100 g D.M.) contained the highest amount of 4-hydroxybenzoic acid, followed sequentially by BR-MG1 (0.881 ± 0.101 g/100 g D.M.), BR-FST2.11 (0.864 ± 0.078 g/100 g D.M.), and BR-FST1.7 (0.861 ± 0.004 g/100 g D.M.) with amounts comparable to BR-UnF and BR-Ster. The lowest amount of 4-hydroxybenzoic acid was recorded in BR-29 (0.653 ± 0.034 g/100 g D.M.). With regards to vanillic acid levels, no significant differences were found between samples as contents ranged between 1.144–1.551 g/100 g D.M., except in the case of BR-FST1.7, which had a lower amount (0.553 ± 0.006 g/100 g D.M.). Phenyllactic acid was not detected in BR-UnF and BR-Ster. Among fermented ingredients, BR-R29 and BR-FST1.7 contained the highest amounts of phenyllactic acid, with values of 15.645 ± 0.589 g/100 g D.M. and 13.387 ± 0.074 g/100 g D.M. quantified, respectively. A significantly lower amount of phenyllactic acid was determined in BR-TR116 (5.254 ± 0.090 g/100 g D.M.) and BR-FST2.11 (5.223 ± 0.740 g/100 g D.M.), with the lowest amount of phenyllactic acid quantified in BR-MG1 (0.562 ± 0.078 g/100 g D.M.). Hydroferulic acid was detected only in BR-MG1 (0.924 ± 0.050 g/100 g D.M.), BR-FST2.11 (0.973 ± 0.132 g/100 g D.M.), and BR-FST1.7 (0.815 ± 0.019 g/100 g D.M.), with no significant differences found between samples. Coumaric acid was quantified in BR-Ster (0.537 ± 0.090 g/100 g D.M.) and BR-TR116 (0.842 ± 0.057 g/100 g D.M.), with the amount quantified in BR-TR116 being significantly higher than the amount determined in BR-Ster. BR-UnF contained 1.008 ± 0.095 g/100 g D.M. of ferulic acid and was the lowest amount detected among ingredients. A significantly higher amount of ferulic acid was noted in BR-Ster (1.656 ± 0.197 g/100 g D.M.). A significant increase in ferulic acid content was measured in BR-TR116 (2.220 ± 0.079 g/100 g D.M.), with the highest amount recorded in BR-R29 (4.068 ± g/100 g D.M.). Ferulic acid was not quantified in BR-MG1, BR-FST2.11, and BR-FST1.7.

##### Bread Fungal Shelf-Life

**Figure 3:**
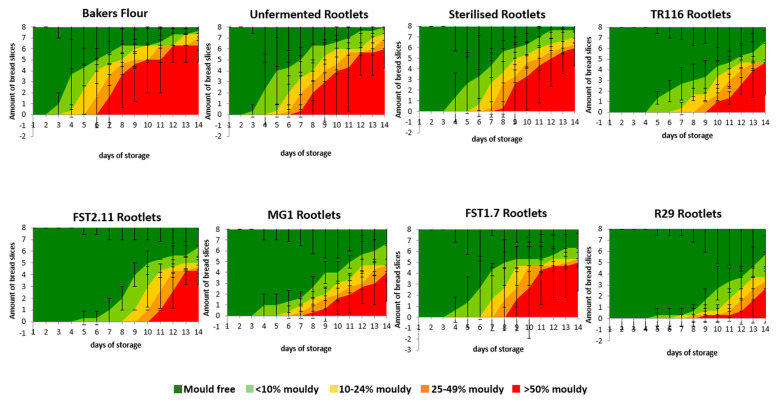
Fungal shelf-life evaluation of bread containing unfermented and fermented BR. TR116, FST2.11, MG1, FST1.7, and R29 denote BR recipes fermented with the strains *Leuconostoc citreum* TR116, *Lactobacillus amylovorus* FST2.11, *Weissella cibaria* MG1, *Lactiplantibacillus plantarum* FST1.7, and *Limosilactobacillus reuteri* R29. The graph represents the means of three independent batches, with error bars representing the standard deviation between batches.

The extent of mould growth on the breadcrumb was analysed to investigate the shelf-life of the bread and determine the kinetics of bread mould growth over time. The results of the microbial shelf-life analysis are illustrated in Figure 3.

The first mould growth on the BF was noted after two days, which was similar to the BR-UnF formulation. Relatively similar kinetics were observed in the BF and BR-UnF recipes. The incorporation of BR-Ster prolonged the microbial shelf-life of the bread by one day, with the kinetics of the mould growth also being slightly slower compared to control recipes. Similar to the BR-Ster recipe, formulations containing BR-MG1 and BR-FST1.7 remained mould-free until day 3. However, the extent of mould growth over time in the BR-MG1 and BR-FST1.7 recipes was much slower. A further increase in the number of mould-free days (+1 day) was observed with BR-TR116 and BR-FST2.11 addition, with a considerable delay in mould growth over time compared to BR-Ster, BR-UnF, and BF controls. The inclusion of BR-R29 had the most substantial effect on microbial shelf-life extension, with no mould growth observed until day 4 and little to no increase in mould growth for up to 8 days.

#### 3.3.6. In Vitro Starch Hydrolysis

**Figure 4:**
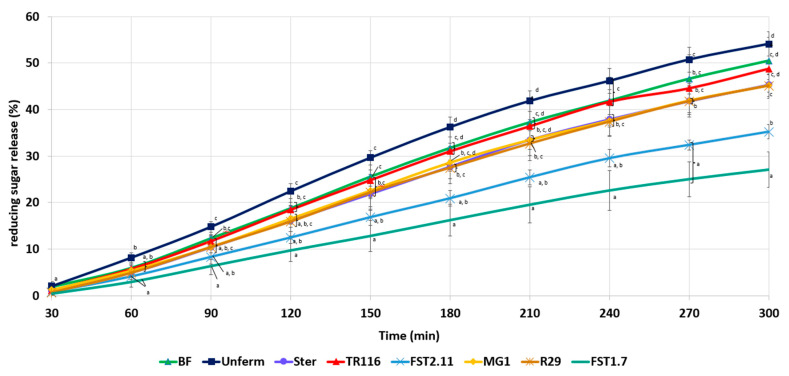
Release of reducing sugars during the in vitro starch hydrolysis of BR breads. BF, UnFerm, and Ster represent control recipes made using baker’s flour and 5% supplementation with unfermented and sterile rootlets, respectively. TR116, FST2.11, MG1, FST1.7, and R29 represent the recipes with 5% replacement of fermented rootlets made using *Leuconostoc citreum* TR116, *Lactobacillus amylovorus* FST2.11, *Weissella cibaria* MG1, *Lactiplantibacillus plantarum* FST1.7, and *Limosilactobacillus reuteri* R29, respectively. The graph illustrates the mean values of triplicate samples with standard deviations represented by error bars. Graph points that share the same letter at each time point do not differ significantly.

The starch digestibility was examined to provide insight into the nutritional characteristics of the bread. The results of the release of reducing sugars (RSR) over time are illustrated in Figure 4.

Bread with supplementation of BR-UnF resulted in the highest RSR curve. A slight reduction in the RSR value was noted in the BF and BR-TR116 bread formulations compared to BR-UnF. A further reduction in RSR release was also observed in BR-Ster, BR-MG1, and BR-R29 compared to BR-UnF, but was not statistically significant in BF and BR-TR116 formulations. Bread formulations with the inclusion of BR-FST2.11 and BR-FST1.7 slowed the release of reducing sugars to the greatest extent, particularly in the case of BR-FST1.7, where the lowest RSR was recorded, which was significantly lower than control formulations BF, BR-UnF, and BR-Ster.

#### 3.3.7. Sensory Analysis of the BR Bread

A sensory analysis of the BR bread was conducted to determine the effect of BR inclusion on the overall sensory experience of the bread and to determine its palatability. Results are represented in Table 7.

Overall, the odour perception of all BR bread was acceptable and comparable to the BF control. No significant differences were found between the bread samples regarding any of the odour attributes. Concerning the taste parameters analysed, some significant differences were found between BR breads. The lowest sourness was perceived in the BR-Ster (0.72 ± 0.83) and BF (0.78 ± 1.26) controls. An increase in sourness was noted in the BR-UnF bread (1.61 ± 1.42); however, this was not significantly different from the BR-Ster and BF formulations. The inclusion of fermented BR increased sourness. The BR-TR116, BR-MG1, and BR-R29 breads had sourness values of 2.17 ± 1.86, 2.22 ± 1.66, and 2.89 ± 2.14, respectively, which were comparable to the BR-UnF formulation but significantly higher than the BF and BR-Ster formulations. An even greater increase in sourness was recorded in BR-FST2.11 (4.22 ± 2.34) and BR-FST1.7 (4.33 ± 2.83), which were significantly higher than all control recipes as well as fermented samples BR-TR116 and BR-MG1. No significant difference was noted in flavour intensity, aftertaste, or the muddy/earthy flavour parameters; however, significant differences were detected between samples for fruity and vegetable flavour compounds in BR bread. Fruity flavours were lowest in BF (0.44 ± 0.51) and BR-Ster (0.61 ± 0.70). An increase in fruity flavours was noted in the BR-UnF (1.56 ± 1.76), BR-TR116 (1.39 ± 1.38), and BR-MG1 (1.56 ± 1.50) compared to the BF and BR-Ster. The values for fruity flavours found in BR-R29 (2.39 ± 1.42) and BR-FST1.7 (2.89 ± 2.49) were slightly higher than those observed in the other fermented samples; however, this was not found to be significant. The highest perception of fruity flavours was detected in the BR-FST2.11 formulations, which were comparable to the BR-R29 and BR-FST1.7 formulations but significantly higher than all control samples. Regarding the vegetable notes in the rootlet breads, BF had the lowest value (1.06 ± 0.94), whereas the inclusion of BR-Ster resulted in the highest perception of vegetable flavours (3.06 ± 1.55). The BR-TR116 addition also resulted in an elevated vegetable flavour that was similar to the BR-Ster sample. Among the remaining samples (BR-UnF, BR-MG1, BR-FST2.11, BR-R29, and BR-FST1.7), no difference in vegetable flavour was detected between samples, with values ranging from 1.89–2.50. Hardness and chewiness values ranged between 3.33–4.00 and 4.50–5.22, respectively, with no significant differences observed between samples. In addition, all breads analysed were ranked highly in overall acceptability (<7), with no significant differences found among the breads tested.

## 4. Discussion

The introduction of food by-products into the food chain has become an important part of research to address various aspects of sustainability goals for the future. Fortification of staple foods with by-products poses difficulties, with deterioration in food prototype quality observed, particularly at higher levels of inclusion [3,5,13,54,55]. Hence, further valorisation and additional processing are required. Neylon et al. [19] demonstrated the significant potential of LAB fermentation to improve BR quality at a fundamental level, while this study illustrates the translation of various benefits imparted by individual LAB fermentations when incorporated into bread prototypes.

The addition of BR, regardless of the processing applied, significantly reduced the strength of the gluten network, suggesting that BR interferes with the formation of the gluten network. The reductions in gluten network strength are likely a contribution of the changes in minerals and protein and fibre interactions with BR addition. BR contain a variety of minerals [3], which could introduce a charge effect and negatively impact the fundamental bonds required for gluten network development [56]. The inclusion of BR also introduces a certain amount of protein to the system, containing a variety of charged amino acids [3], which can also interfere with the intramolecular gluten network bonds. Finally, fibre constitutes a large proportion of the BR composition, which has been seen to alter gluten development and techno-functional characteristics, which may contribute to the negative defects observed [57,58]. Fibres have been shown to create a physical hindrance in gluten development, negatively interfere with the hydration of the gluten network, and facilitate gluten-fibre interactions that result in weakened gluten techno-functional characteristics [57,58]. The inclusion of fermented BR reduced the amount of time taken for the gluten network to develop, which might be due to enhanced hydrophobic interactions. The inclusion of a fermented ingredient putatively creates a more acidic environment, which may change the configuration of proteins, further exposing the hydrophobic regions of proteins [26,37]. This might amplify hydrophobic interactions during gluten development, which is believed to stabilise gluten development [59], thus reducing the amount of time required for the network to form. In addition to this, fermentation of BR increases mineral bioavailability [60], resulting in a possible charge screening effect that may further help expose apolar amino acid chains and encourage hydrophobic interactions to a greater extent.

The time taken for gluten network development also correlated positively (r = 0.899, *p* < 0.05) to the damping factor from rheological measurements, indicating that the time taken for gluten network development was influential in determining viscoelastic properties. The inclusion of BR-UnF maintained the same viscoelastic properties as BF, which could be linked with the alpha-amylase activity of BR-UnF. Alpha amylases degrade starch molecules into small chain dextrins, which reduce dough viscosity [61] and thus likely increase the viscous parts of the dough. Moreover, proteases play a vital role in the germination stage of malting [62,63], which is where BR is sourced from. Thus, BR-UnF likely contains natural proteases, which may also induce a dough-softening effect and enhance the viscous parts of the dough [64,65]. In contrast, BR-Ster increased the elastic (solid) proportions of the dough. This was likely due to the elimination of the enzymatic effects associated with BR-UnF with the heat treatment applied and the overall reduction in starch and gluten within the matrix of the dough with flour replacement, resulting in a dough with enhanced resistance to deformation. For fermented formulations, the enhanced hydrophobic interactions discussed previously may putatively stabilise the gluten network development in the dough matrix, resulting in a dough with greater resistance to deformation. The greater increase in elastic parts in BR-FST2.11 and BR-FST1.7 compared to the other fermented ingredients could be due to the greater acid contents present in these ingredients [19]. This may enhance the amount of positive charges present, facilitating hydrophobic interactions during gluten development [66] and producing a stiffer dough.

The DF also influenced the maximum dough rise (Hm) achieved during the yeast fermentation (r = 0.885, *p* < 0.05), indicating that viscoelastic properties facilitate dough expansion during the fermentation. Interestingly, the BR-MG1 formulation achieved a high Hm, which may be explained by the exopolysaccharides present in the BR-MG1 ingredient after fermentation. *W. cibaria* MG1 is capable of producing exopolysaccharides of the dextran type and gluco-oligosaccharide type from sucrose [36,39], which can be used to enhance bread structure and mimic the behaviour of hydrocolloids [67]. A previous study investigating the use of *W. cibaria* MG1 in sourdough attributed the enhanced loaf volume and reduced crumb hardness to the dextran exopolysaccharides [68]. Thus, higher Hm values during proofing could be achieved through a reinforced dough structure with the presence of exopolysaccharides in BR-MG1. Galle et al. [68] also observed increased production of CO_2_ with the inclusion of *W. cibaria* MG1 in sourdough, suggesting the increased production was a result of an enhanced level of sugars present in the sourdough, favouring yeast fermentation. Results from Vtotal CO_2_ values in this study complement these findings. The BR-MG1 ingredient (as well as BR-FST2.11 and BR-FST1.7) contains higher levels of residual glucose and fructose [19], which could enhance yeast metabolism and increase the volume of CO_2_ produced during proofing. However, it is important to note that the inclusion of BR regardless of the processing applied enhanced CO_2_ production, indicating BR did not interfere negatively with yeast metabolism but rather improved it. This suggests BR is likely a suitable substrate for inclusion in yeast-leavened products. Thus, the adverse effects on dough rise observed are likely more connected to the observations made in DF values previously discussed. However, in contrast to this trend was the BR-R29 formulation, which behaved differently during the yeast fermentation with a reduced amount of CO_2_ produced. This could be linked to the levels of antifungals present (discussed in later sections) as well as the probable reuterin present [42] in the ingredient, which inhibits yeast metabolism and thus dough expansion to a certain extent. However, volumes of CO_2_ produced were relatively similar to amounts produced in the BF control, indicating that the negative impacts observed were not of major significance.

Hm values correlated positively to bread specific volume (r = 0.90, *p* < 0.05), indicating the height reached during yeast fermentation affected bread volumes. BR-MG1 bread had an improved specific volume, reaching volumes higher than those observed for the BF wheat control, suggesting fermentation of BR with *W. cibaria* MG1 had a positive influence on bread quality. The enhancement observed can be attributed to the more stabilised dough structure due to the presence of exopolysaccharides. Exopolysaccharides have been described to perform similarly to hydrocolloids in a bread system [69] and increase bread-specific volumes [70]. The inclusion of BR-TR116 also maintained the bread-specific volume, which was comparable to BF. Although *L. citreum* TR116 can also produce exopolysaccharides [28], the presence of exopolysaccharides was unlikely as the fermentation was supplemented with fructose, which facilitates the production of mannitol and acetate [19]. Higher volumes of acetate have been reported in the literature to negatively influence dough extensibility and volume [71], but this did not appear to negatively impact the BR-TR116 bread quality. This might be linked to the addition level of the ingredient, which may not have reached the threshold for this hypothesis to take effect. Incorporation of BR-UnF also achieved specific volumes comparable to the BF, likely due to the similarities observed in viscoelastic properties as well as the enhanced yeast metabolism. The lower specific volumes with the inclusion of BR-Ster, BR-FST2.11, BR-R29, and BR-FST1.7 could be associated with the higher elastic properties of these doughs, restricting dough rise. The higher acidification effect in the FST1.7 and FST2.11 formulations may have had an extra contributory effect and may constitute a reason for the lowest volumes observed in these formulations. In the case of BR-R29, the reductions observed in bread-specific volume coincide and can be explained by inhibited yeast fermentation. The variations observed in crumb hardness may be attributed to the differences noted in bread specific volume, as crumb hardness and bread specific volume had a strong negative correlation (r = −0.957, *p* < 0.01). The addition of BR-UnF produced a soft crumb, likely due to the alpha-amylase activity present in BR-UnF [19], which has a positive influence on crumb texture [72]. The inclusion of BR ingredients fermented with *W. cibaria* MG1 and *L. citreum* TR116 also positively influenced crumb hardness, as even lower crumb hardness (compared to BF) was observed, highlighting further enhancements to bread quality using BR-MG1 and BR-TR116 inclusion. Although variations in crumb hardness were observed, overall, relatively similar trends in crumb resilience suggest inclusion of BR does not negatively impact the crumb’s integrity. Dough characteristics such as Hm and DF also had an impact on crumb cell structure, with significant positive correlations observed with cell diameter values (r = 0.97, < 0.05, and r = 0.91, *p* < 0.05, respectively). Thus, variations in cell diameters observed with BR inclusion are a result of the restrictions imposed on the viscoelastic properties constricting the cell diameter size.

Aside from gluten network development and dough rheological analysis, in-depth analysis of the effects on gluten and starch pasting during mixing and heating provided by Mixolab analysis gives further insight into bread quality. The inclusion of BR increased dough development times, likely due to the higher amount of fibre inclusion in bread formulations, which has previously been shown to extend dough development times [50,73,74]. This may be due to the increase in competition for water with fibre inclusion [70,75], as BR-UnF and BR-Ster have an exceptionally high water binding capacity [19]. The addition of fermented BR ingredients reduced the amount of time required for the dough to form. This may be linked to the reduced water-binding capacity of the fermented ingredients [19], which would likely reduce the competition for hydration. In addition, the reductions in dough development times with the inclusion of fermented ingredients align with the shorter gluten network development times seen previously, indicating optimal dough development occurs at an earlier stage with fermented ingredient inclusion. Thus, as fully developed doughs reflect optimal dough quality, results from this study suggest alterations in dough mixing times during the baking process may further enhance BR bread dough quality. The reductions in C2 values suggest a protein weakening effect [49] with BR inclusion and complement the results observed previously with decreased gluten network strength. The greatest reductions in C2 observed with BR-UnF, BR-FST2.11, and BR-FST1.7 suggest that the protein destabilisation effect was process-dependent. The protein weakening effect with BR-UnF could be a result of active proteases in BR-UnF, which can weaken the integrity of the gluten network [37]. The enhanced protein weakening effect of BR-FST2.11 and BR-FST1.7 might be linked to the higher acid content of these BR ingredients [19]. The higher acid content, which as previously stated may enhance the formation of the gluten network, could also activate some proteolytic activity in the wheat flour [76], resulting in weakening the integrity and functionality of the gluten network over time. Reductions in C3 values indicate lower levels of starch swelling. The reduction in starch swelling with BR inclusion was to be expected with the inclusion of a fibre/protein rich material such as BR, which limits the amount of starch available to contribute to this reaction [9,10,75]. The more pronounced reduction in starch swelling in BR-UnF reflects the high alpha-amylase activity of the unfermented BR ingredient [19], which adversely effects starch granule integrity [65], hence negatively impacting its hydration properties. The higher enzymatic activity hypothesis can also be applied to the rupturing of starch granules (C4 values) and starch retrogradation properties (C5 values) of the BR-UnF dough. Higher alpha amylase activity accumulates low molecular weight dextrin and thus reduces viscosity, which explains the lower C4 values [77]. Furthermore, higher alpha amylase activities restrict realignment during the retrogradation process with increasing low molecular weight dextrins [78,79]. The minor differences observed with BR ingredient inclusion across all other formulations indicate no major differences were detected in the starch pasting properties, indicating little strain dependency effects.

Observations from microbial shelf-life kinetics show inclusion of BR-UnF resulted in a microbial shelf-life similar to that of BF, indicating BR-UnF did not influence the microbial shelf-life. The one-day increase in microbial shelf-life with BR-Ster addition illustrates how the sterilisation process mitigates some natural microflora present in the BR-Ster ingredient, which may help to extend the microbial shelf-life. The further extension in microbial shelf-life (+1 day) with fermented BR addition can be linked with the antifungal metabolites imparted on the BR ingredients during fermentation. Sourdough technology with numerous LAB strains has been previously shown to provide a natural antimicrobial effect due to the synergistic effect of the variety of antifungal metabolites and organic acids produced during fermentation [41,80]. BR-MG1 had the lowest amount and variety of antifungal compounds present, suggesting *W. cibaria* MG1 does not give an enhanced antifungal effect and explaining the similar shelf-life kinetics observed with BR-Ster inclusion. Among the fermented formulations, BR-R29 provided the most significant reduction in microbial growth kinetics due to the highest amount/variety of antifungal compounds and their synergistic effect with lactic and acetic acids, creating an even greater hurdle effect [41]. Previous studies using *L. reuteri* R29 found success in microbial shelf-life extension in bread and attributed this primarily to the exceptionally high levels of phenyllactic acid produced during fermentation [41,42], complementing the results found in this study. *L. amylovorus* FST2.11, *L. plantarum* FST1.7, and *L. citreum* TR116 have also been documented as antifungal producers across a variety of substrates [26,30,32,44,81]. In this study, the low BR addition level may have limited their potency as a natural shelf-life extender. Thus, fermentation of BR with *L. reuteri* R29 and their addition into bread at 5% inclusion may aid in the formulation of clean-label bread products and reduce the level of chemical preservatives such as propionate or sorbate in formulations [82,83].

Due to the high fibre content of the BR ingredient, its incorporation into a bread formulation will enhance the fibre content of the bread [5], thus enhancing the nutritional benefits of the bread product [84]. However, analysis of changes in the extent of sugar release over time from bread delves further into the potential nutritional benefits of BR inclusion. BR-UnF did not slow sugar release over time but increased the amount of sugar released from the bread matrix. This might be due to the enhanced enzymatic activity of BR-UnF, which favours the hydrolysis of starch during proofing and increases the amount of readily available sugars capable of being released from the bread matrix. This also explains the slightly lower sugar release observed with BR-Ster inclusion, as the enzymatic activity influence was eliminated post-sterilisation. The reductions observed with BR-Ster, BR-TR116, BR-R29, and BR-MG1 are likely due to the overall reduction in starch available for hydrolysis with the replacement of a fibre ingredient. Interestingly, BR-FST2.11 and BR-FST1.7 showed a different trend, and a notably lower sugar release curve over time was observed. The inclusion of fermented by-products has previously been shown to inhibit starch hydrolysis in bread [9,14,16,18], which in this study appears to be a strain-dependent characteristic. The reduction in the level of starch hydrolysis may be attributed to the much higher amounts of lactic acid in these ingredients [19], which negatively interfere with starch hydrolysis. Studies from Östman et al. [85] show the presence of lactic acid during heat treatment encourages interactions between starch and gluten, which limits the starch available for hydrolysis. Thus, fermentation of BR with *L. plantarum* FST1.7 and *L. amylovorus* FST2.11 might be more beneficial for the engineering of products for consumers with diabetes. The higher levels of acid in BR-FST1.7 and BR-FST2.11 could also have led to changes observed in the sensory perception of these bread prototypes, with enhancements noted in sour taste and fruity flavours. Increases in fruity and sour tastes are typically found in wheat sourdough bread [86], indicating a sourdough-like bread flavour may be achieved through fermentation of BR by *L. plantarum* FST1.7 and *L. amylovorus* FST2.11. The high comparability of BF bread and BR breads across all sensory attributes indicates that a 5% BR inclusion level in a bread system is highly acceptable and complements the bread sensory results previously seen [3,5].

## 5. Conclusions

To date, the inclusion of BRs and investigation of their effects in bread making have been limited. However, this study shows a promising future for BR in the baking industry, particularly in yeast-leavened products. Furthermore, upcycling processes such as LAB fermentation prove to be viable processing tools for BR, with unique fermentation characteristics being translated to the bread matrix. The addition of BR into the bread system at a 5% addition level was found to decrease the strength of the gluten network, however, the introduction of the fermented ingredients amplified the speed required for gluten development. Concerning the unfermented BR formulation, positive impacts were also observed in the viscoelastic properties of the dough, which were mainly attributed to the enzymatic activity of the unprocessed ingredient. The addition of the BR-MG1 and BR-TR116 ingredients showcased their potential to improve bread quality with high specific volumes and softer crumbs. Moreover, the inclusion of BR-R29 illustrates its power to reduce the microbial growth rate to a significant extent through the natural production of high amounts of antifungal compounds. The incorporation of BR-FST1.7 and BR-FST2.11 shows promising potential to further improve the nutritional characteristics of the BR with a slower release of sugars over time during starch digestion. Furthermore, the inclusion of BR-FST1.7 and BR-FST2.11 altered the sensory experience of wheat bread, creating a bread with flavours that might compare more similarly to those of conventional sourdough. Hence, this study showcases how the LAB fermentation of BR can be tailored based on desired requirements in the bread application at a later stage. Thus, future work in this area indicates that a co-fermentation of BR with multifunctional LAB starter cultures might functionalise BR to an even greater extent and endorse its inclusion in leavened cereal products.

## Figures and Tables

**Table 1:** Characteristics of lactic acid bacteria incorporated in barley rootlet ingredient development.

Species	*Leuconostoc* *citreum*	*Lactobacillus amylovorus*	*Weissella* *cibaria*	*Limosilactobacillus reuteri*	*Lactiplantibacillus plantarum*
Strain	TR116	FST 2.11	MG1	R29	FST 1.7
Metabolism	Heterofermentative	Homofermentative	Heterofermentative	Heterofermentative	Heterofermentative
Fermentation substrate	Fructose	Sucrose	Sucrose	Sucrose	Sucrose
Source	Yellow pea sourdough	Brewing environment	Sourdough	Human intestine	Malted barley
Special traits	Mannitol producer and antifungal producer	Antimicrobial producer and high acid producer	Dextran exopolysaccharide producer	Mannitol producer and antifungal producer	Antifungal producer and high acid producer
Reference	[26,27,28,29,30,31]	[32,33,34]	[35,36,37,38,39,40]	[41,42,43]	[34,43,44,45,46]

**Table 2:** Compositional analysis of bakers’ flour (BF) and unfermented barley rootlets (UnF-BR) measured in g/100 g. Results are represented as mean values ± standard deviations.

Analyte	BF	BR-UnF
Protein	12.96 ± 0.79	35.80 ± 1.50
Fat	1.20 ± 0.08	1.77 ± 0.11
Ash	0.55 ± 0.05	5.98 ± 0.30
Moisture	12.95 ± 0.30	12.74 ± 0.30
Total Carbohydrate	65.31 ± 1.53	0 < 7.02 < 15.72
Total fibre	7.03 ± 1.27	36.64 ± 8.51
Soluble fibre	2.63 ± 0.63	1.24 ± 0.30
High molecular weight of dietary fibre	4.41 ± 1.1	35.40 ± 8.50

**Table 3:** Recipes for bread preparation expressed as a % based on flour, which equates to the sum of the baker’s flour (BF) and barley rootlet (BR) ingredients. UnF and Ster represent unfermented and heat-sterilised barley rootlets, respectively. BR-TR116, BR-MG1, BR-FST2.11, BR-R29, and BR-FST1.7 express barley rootlets fermented with their respective LAB strains, namely *Leuconostoc citreum* TR116, *Weisella cibaria* MG1, *Lactobacillus amylovorus* FST2.11, *Limosilactobacillus reuteri* R29, and *Lactiplantibacillus plantarum* FST1.7, respectively. FWA % represents results obtained from farinograph water absorption, with letters sharing the same subscript numbers and not significantly differing (*p* < 0.05).

Ingredient	BF	BR-UnF	BR-Ster	BR-TR116	BR-MG1	BR-FST2.11	BR-R29	BR-FST1.7
BF	100	95	95	95	95	95	95	95
BR ingredient	-	5	5	5	5	5	5	5
Salt	1.2	1.2	1.2	1.2	1.2	1.2	1.2	1.2
Sugar	2.0	2.0	2.0	2.0	2.0	2.0	2.0	2.0
Sunflower oil	3.2	3.2	3.2	3.2	3.2	3.2	3.2	3.2
Dry Yeast	2.0	2.0	2.0	2.0	2.0	2.0	2.0	2.0
Water (FWA%)	64.3 ^a^	68.2 ^c,d^	68.9 ^d^	66.90 ^b^	67.90 ^c^	67.50 ^b,c^	66.70 ^b^	67.30 ^b,c^

**Table 4:** Dough characteristics with BR inclusion. BF, BR-UnF, and BR-Ster represent control recipes using baker’s flour, 5% unfermented barley rootlet inclusion, and 5% sterilised, freeze-dried barley rootlet inclusion, respectively. BR-TR116, BR-MG1, BR-FST2.11, BR-R29, and BR-FST1.7 denote recipes including fermented rootlets at a 5% addition level and fermented using *L. citreum* TR116, *W. cibaria* MG1, *L. amylovorus* FST2.11, *L. reuteri* R29, and *L. plantarum* FST1.7, respectively. Results are illustrated as mean values ± standard deviations. Samples in the same row that share the same subscript letter had no significant statistical differences (*p* < 0.05).

	BF	BR-UnF	BR-Ster	BR-TR116	BR-MG1	BR-FST2.11	BR-R29	BR-FST1.7
Torque Max (BU)	78.33 ± 1.54 ^c^	64.00 ± 1.00 ^a,b^	61.67 ± 2.53 ^a,b^	60.33 ± 4.04 ^a,b^	62.333 ± 1.53 ^a,b^	65.33 ± 1.16 ^b^	58.67 ± 1.54 ^a^	60.00 ± 1.00 ^a^
Peak Max Time (s)	39.67 ± 1.53 ^c^	44.00 ± 1.00 ^c^	40.00 ± 2.00 ^c^	33.67 ± 3.22 ^b^	32.67 ± 2.08 ^b^	25.67 ± 1.16 ^a^	32.33 ± 0.58 ^b^	26.67 ± 1.16 ^a^
DDT (min)	1.32 ± 0.27 ^a^	7.04 ± 0.23 ^d^	7.49 ± 0.32 ^d^	5.61 ± 0.07 ^c^	4.95 ± 1.00 ^a,b,c,d^	4.21 ± 0.03 ^b^	3.66 ± 0.15 ^b^	3.87 ± 0.10 ^b^
C2 (Nm)	0.43 ± 0.01 ^g^	0.33 ± 0.00 ^b,c^	0.38 ± 0.00 ^f^	0.35 ± 0.01 ^d,e^	0.36 ± 0.00 ^e^	0.31 ± 0.01 ^a,b^	0.34 ± 0.00 ^c,d^	0.30 ± 0.01 ^a^
C3 (Nm)	1.63 ± 0.01 ^d^	1.24 ± 0.02 ^a^	1.52 ± 0.01 ^b^	1.56 ± 0.02 ^b,c^	1.53 ± 0.00 ^b^	1.63 ± 0.00 ^c,d^	1.60 ± 0.01 ^c,d^	1.62 ± 0.01 ^c,d^
C4 (Nm)	1.50 ± 0.01 ^e,f^	0.71 ± 0.03 ^a^	1.38 ± 0.01 ^b^	1.44 ± 0.01 ^c,d^	1.41 ± 0.01 ^b,c^	1.51 ± 0.01 ^f^	1.47 ± 0.01 ^d,e^	1.50 ± 0.01 ^e,f^
C5 (Nm)	2.55 ± 0.03 ^d^	1.12 ± 0.03 ^a^	2.23 ± 0.03 ^b^	2.34 ± 0.02 ^c^	2.28 ± 0.01 ^b,c^	2.65 ± 0.03 ^e^	2.59 ± 0.05 ^d,e^	2.61 ± 0.02 ^d,e^
Hm (mm)	50.93 ± 2.48 ^c^	46.40 ± 1.31 ^b,c^	40.43 ± 0.57 ^a,b^	43.17 ± 3.26 ^b^	44.87 ± 1.52 ^b,c^	37.00 ± 0.30 ^a^	36.57 ± 2.53 ^a^	34.97 ± 3.10^a^
Vol. of CO_2_ (mL)	2045 ± 73^a,b,c^	2451 ± 30^d^	2399 ± 100^d^	2029 ± 69^a,b^	2547 ± 68^d^	2318 ± 131^c,d^	1919 ± 59^a^	2302 ± 173 ^b,c,d^
CO_2_ retention coefficient (%)	97.20 ± 3.33 ^a^	99.63 ± 0.12 ^a^	99.70 ± 0.00 ^a^	99.57 ± 0.15 ^a^	99.73 ± 0.12 ^a^	99.67 ± 0.06 ^a^	99.60 ± 0.17 ^a^	99.73 ± 0.06 ^a^
Damping factor	0.390 ± 0.012 ^d^	0.383 ± 0.009 ^d^	0.357 ± 0.010 ^c^	0.346 ± 0.012 ^a,b,c^	0.352 ± 0.011 ^b,c^	0.328 ± 0.006 ^a^	0.344 ± 0.011 ^a,b,c^	0.333 ± 0.014 ^a,b^

**Table 5:** Bread quality characteristics with the inclusion of barley rootlets and novel fermented barley rootlet ingredients (5% inclusion, based on flour). BF represents a control recipe using only baker’s flour. BR-UnF and BR-Ster denote control recipes using unfermented barley rootlets and sterile, freeze-dried barley rootlets, respectively. BR-TR116, BR-MG1, BR-FST2.11, BR-R29, and BR-FST1.7 illustrate fermented rootlet recipes with ingredients fermented using *L. citreum* TR116, *W. cibaria* MG1, *L. amylovorus* FST2.11, *L. reuteri* R29, and *L. plantarum* FST1.7, respectively. Results are represented as means ± standard deviation. No significant differences were found between samples that shared the same subscript letter in the same row (*p* < 0.05).

	BF	BR-UnF	BR-Ster	BR-TR116	BR-MG1	BR-FST2.11	BR-R29	BR-FST1.7
Predicted fibre content (g/100 g)	4.74	5.57	5.55	5.66	5.63	5.61	5.61	5.60
Digestible starch content (g/100 g)	40.61 ± 1.46 ^c^	38.68 ± 0.80 ^a^	35.64 ± 0.56 ^a,b^	36.80 ± 1.14 ^a,b^	37.17 ± 0.66^b^	36.11 ± 1.02 ^a,b^	36.32 ± 0.76 ^a,b^	36.85 ± 0.73 ^a,b^
Bake loss (%)	14.24 ± 0.91 ^a,b,c,d,e^	13.54 ± 0.58 ^b,c,e^	13.56 ± 0.53 ^b,c^	14.33 ± 0.47 ^d,e^	14.19 ± 0.38 ^a,d,e^	13.85 ± 0.31 ^a,b,c,d,e^	13.36 ± 0.58 ^a,b,c^	13.54 ± 0.93 ^a,b,c,d,e^
Specific volume (mL/g)	3.74 ± 0.20 ^d,e^	3.55 ± 0.14 ^c,d^	3.09 ± 0.18 ^b^	3.68 ± 0.22 ^c,d,e^	3.80 ± 0.13 ^e^	2.80 ± 0.15 ^a^	3.35 ± 0.29 ^b,c^	2.63 ± 0.19 ^a^
Cell diameter (mm)	2.93 ± 0.15 ^e^	2.16 ± 0.15 ^d^	1.94 ± 0.18 ^b,c^	1.95 ± 0.17 ^b,c^	2.05 ± 0.16 ^c,d^	1.83 ± 0.22 ^b^	1.83 ± 0.15 ^b^	1.63 ± 0.16 ^a^
Breadcrumb hardness (N)	2.33 ± 0.32 ^c^	1.77 ± 0.26 ^a,b^	3.14 ± 0.42 ^d^	1.96 ± 0.30 ^b^	1.61 ± 0.22 ^a^	3.59 ± 0.53 ^d^	2.39 ± 0.35 ^c^	4.11 ± 0.55 ^d^
Breadcrumb resilience (N)	0.56 ± 0.01 ^c,d^	0.53 ± 0.02 ^b^	0.55 ± 0.01 ^b,c^	0.55 ± 0.02 ^b,c^	0.56 ± 0.01 ^d^	0.52 ± 0.02 ^a^	0.54 ± 0.01 ^b^	0.53 ± 0.01 ^a^

**Table 6:** Analysis of the antifungal compounds present in BR ingredients in g/100 g of dry matter. BR-UnF and BR-Ster represent control ingredients: unfermented barley rootlets and sterile, freeze-dried barley rootlets, respectively. BR-TR116, BR-MG1, BR-FST2.11, BR-R29, and BR-FST1.7 illustrate fermented rootlet ingredients that were fermented using *L. citreum* TR116, *W. cibaria* MG1, *L. amylovorus* FST2.11, *L. reuteri* R29, and *L. plantarum* FST1.7, respectively. Results are represented as means ± standard deviation. No significant differences were found between samples that shared the same subscript letter in the same row (*p* < 0.05).

Antifungal Compound	BR-UnF	BR-Ster	BR-TR116	BR-MG1	BR-FST2.11	BR-R29	BR-FST1.7
Hydroxyphenyllactic acid	n.d.	n.d.	0.637 ± 0.091 ^a^	n.d.	2.483 ± 0.247 ^b^	2.239 ± 0.087 ^b^	9.136 ± 0.104 ^c^
4-Hydroxybenzoic acid	0.928 ± 0.006 ^d^	1.215 ± 0.109 ^c,d^	1.177 ± 0.036 ^c^	0.881 ± 0.101 ^a,c,d^	0.864 ± 0.078 ^a,b,d^	0.653 ± 0.034 ^a^	0.861 ± 0.004 ^b,c^
Vanillic acid	1.316 ± 0.018 ^b^	1.551 ± 0.171 ^b^	1.270 ± 0.038 ^b^	1.367 ± 0.150 ^b^	1.144 ± 0.157 ^a,b^	1.199 ± 0.057 ^b^	0.553 ± 0.006 ^a^
Phenyllactic acid	n.d.	n.d.	5.254 ± 0.179 ^b^	0.562 ± 0.078 ^a^	5.223 ± 0.740 ^b^	15.645 ± 0.589 ^c^	13.387 ± 0.074 ^c^
Hydroferulic acid	n.d.	n.d.	n.d.	0.924 ± 0.050 ^a^	0.973 ± 0.132 ^a^	n.d.	0.815 ± 0.019 ^a^
Coumaric acid	n.d.	0.537 ± 0.090 ^a^	0.842 ± 0.057 ^b^	n.d.	n.d.	n.d.	n.d.
Ferulic acid	1.008 ± 0.095 ^a^	1.656 ± 0.197 ^b^	2.220 ± 0.079 ^c^	n.d.	n.d.	4.068 ± 0.164 ^d^	n.d.

n.d. defined as not detected.

**Table 7:** Descriptive sensory analysis of BR breads. BF represents results obtained from bakers’ wheat flour breads. BR-UnF and BR-Ster denote control recipes made with 5% inclusion of unfermented and sterile rootlets, respectively. BR-TR116, BR-MG1, BR-FST2.11, BR-R29, and BR-FST1.7 illustrate sensory results from bread recipes made with a 5% addition of fermented rootlets produced using *L. citreum* TR116, *W. cibaria* MG1, *L. amylovorus* FST2.11, *L. reuteri* R29, and *L. plantarum FST1.7*, respectively. Results are represented as mean values ± standard deviations. Values in the same row that share the same subscript letter are not significantly different (*p* < 0.05).

	BF	BR-UnF	BR-Ster	BR-TR116	BR-MG1	BR-FST2.11	BR-R29	BR-FST1.7
Odour								
Intensity	5.78 ± 2.21 ^a^	6.39 ± 1.85 ^a^	6.67 ± 2.28 ^a^	6.33 ± 2.11 ^a^	6.78 ± 1.83 ^a^	6.67 ± 2.11 ^a^	6.22 ± 1.86 ^a^	6.22 ± 2.07 ^a^
Citrus	1.67 ± 1.46 ^a^	2.06 ± 1.26 ^a^	1.50 ± 1.86 ^a^	1.72 ± 1.36 ^a^	1.83 ± 0.99 ^a^	2.56 ± 1.65 ^a^	2.17 ± 1.34 ^a^	2.78 ± 1.80 ^a^
Vegetable	1.28 ± 1.27 ^a^	2.28 ± 1.49 ^a^	2.44 ± 1.50 ^a^	1.83 ± 0.99 ^a^	1.78 ± 1.44 ^a^	2.44 ± 1.38 ^a^	2.06 ± 1.70 ^a^	1.67 ± 1.14 ^a^
Cereals/grains	6.06 ± 2.36 ^a^	7.11 ± 1.32 ^a^	7.28 ± 1.36 ^a^	7.00 ± 1.41 ^a^	6.83 ± 1.34 ^a^	6.06 ± 1.30 ^a^	6.06 ± 1.43 ^a^	6.56 ± 1.54 ^a^
Taste								
Sour	0.78 ± 1.26 ^a^	1.61 ± 1.42 ^a,b^	0.72 ± 0.83 ^a^	2.17 ± 1.86 ^b^	2.22 ± 1.66 ^b^	4.22 ± 2.34 ^c^	2.89 ± 2.14 ^b,c^	4.33 ± 2.83 ^c^
Flavour								
Intensity	4.39 ± 1.61 ^a^	5.72 ± 1.64 ^a^	5.67 ± 2.11 ^a^	5.44 ± 1.85 ^a^	5.11 ± 2.05 ^a^	6.22 ± 1.77 ^a^	5.94 ± 1.95 ^a^	6.17 ± 1.95 ^a^
Muddy/earthy	0.89 ± 1.02 ^a^	2.22 ± 2.37 ^a^	2.89 ± 2.37 ^a^	2.50 ± 2.38 ^a^	1.67 ± 1.81 ^a^	2.17 ± 2.33 ^a^	1.83 ± 2.20 ^a^	1.44 ± 1.25 ^a^
Fruity	0.44 ± 0.51 ^a^	1.56 ± 1.76 ^b,c^	0.61 ± 0.70 ^a,b^	1.39 ± 1.38 ^b,c^	1.56 ± 1.50 ^b,c^	3.56 ± 2.45 ^d^	2.39 ± 1.42 ^c,d^	2.89 ± 2.49 ^c,d^
Vegetable	1.06 ± 0.94 ^a^	2.39 ± 1.46 ^a,b^	3.06 ± 1.55 ^b^	2.61 ± 1.65 ^b^	2.39 ± 1.58 ^a,b^	2.50 ± 1.47 ^a,b^	2.50 ± 1.86 ^a,b^	1.89 ± 1.41 ^a,b^
Aftertaste	1.28 ± 1.74 ^a^	2.72 ± 2.44 ^a^	2.94 ± 2.21 ^a^	2.22 ± 1.96 ^a^	2.11 ± 1.64 ^a^	3.39 ± 2.66 ^a^	3.06 ± 2.41 ^a^	3.17 ± 2.36 ^a^
Texture								
Hardness	4.00 ± 2.28 ^a^	3.44 ± 2.28 ^a^	3.83 ± 1.95 ^a^	3.44 ± 1.62 ^a^	3.50 ± 1.76 ^a^	3.89 ± 1.45 ^a^	3.33 ± 1.71 ^a^	3.61 ± 1.65 ^a^
Chewiness	4.50 ± 2.23 ^a^	4.67 ± 2.11 ^a^	4.50 ± 2.01 ^a^	4.78 ± 1.77 ^a^	5.22 ± 2.07 ^a^	4.94 ± 1.63 ^a^	4.56 ± 1.98 ^a^	4.94 ± 1.83 ^a^
Overall acceptability	8.17 ± 1.72 ^a^	7.56 ± 1.82 ^a^	7.33 ± 2.11 ^a^	7.67 ± 1.64 ^a^	8.11 ± 1.41 ^a^	7.28 ± 1.41 ^a^	7.50 ± 1.98 ^a^	7.44 ± 1.46 ^a^

## Data Availability

The data are contained within the text.

## Supplementary Materials

The following supporting information can be downloaded at: https://www.mdpi.com/article/10.3390/foods12071549/s1, Figure S1: Questionnaire used for sensory analysis.
