# Supplementary material for: From Waste to Taste: Application of Fermented Spent Rootlet Ingredients in a Bread System
Source: Foods. 2023 Apr 6;12(7):1549. doi: 10.3390/foods12071549 (PMC10094320; doi:10.3390/foods12071549)
Supplement: Supplementary file 1 [file foods-12-01549-s001.zip › foods-2291387-supplementary updated.pdf]

Descriptive Sensory Evaluation

Personal Data

Name: …………………………………… Date: …………………………………….

Information

- The samples are bread samples with unfermented, sterilised and a variety of different strains of lactic acid bacteria fermented rootlets. Traces of the following allergens may be present: **gluten**, peanuts, tree nuts, celery, mustard, eggs, milk, sesame, fish, lupin, soya, sulphites, crustaceans, molluscs.
- All information provided is confidential and the results will not be associated with your name.
- The products have been produced under safe and food grade conditions.

Please confirm that (tick the circle)

- O You are aware that the participation in this sensory evaluation is voluntary.
- O You are aware that products may contain the allergens reported above.

Guidelines

There are 8 samples to be evaluated. Samples will be provided to you one at a time.

Please smell and taste the samples and estimate the intensity of each of the attributes by placing a line on the scale as shown in the example below. **Please place your making on a line provided.** The attributes and their descriptions are listed in the accompanying Table 1. Adding comments is optional.

In between each sample, please cleanse your palate with water.

Example:

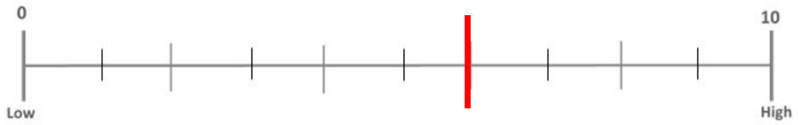

Sample:

Odour

Overall intensity

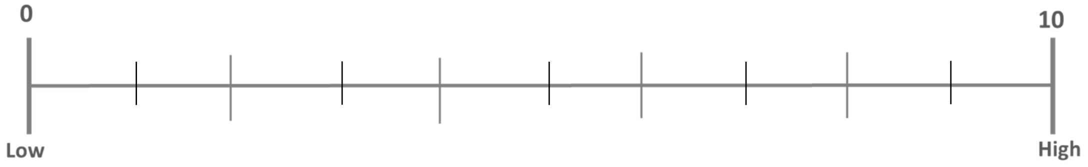

Citrus fruit

0 — Low   10 — High

Vegetable

0 — Low   10 — High

Cereal/grains

0 — Low   10 — High

Comments:

____

____

____

Taste

Sour

0 — Low   10 — High

Sweet

0 — Low   10 — High

Salty

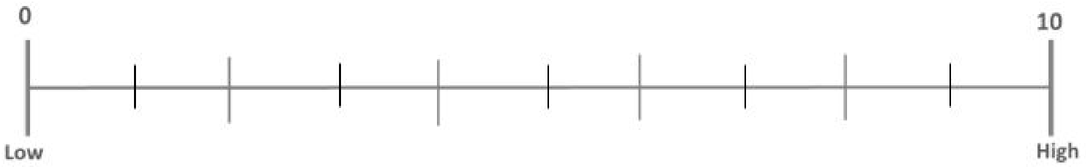

Comments:

____

____

____

Flavour

Overall intensity

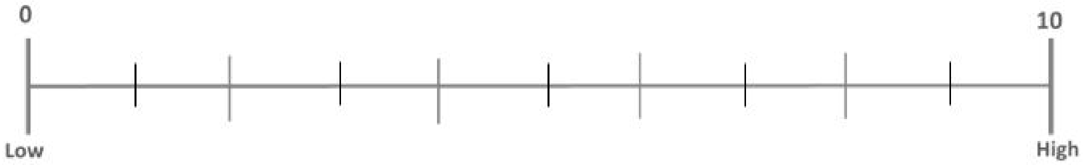

Muddy/Earthy

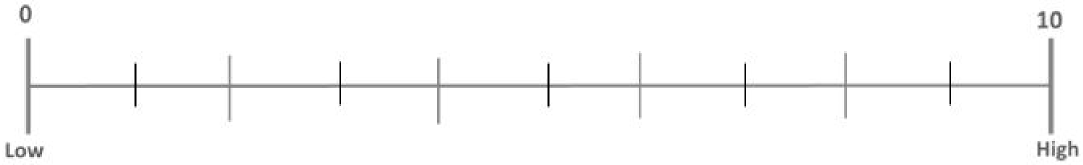

Dairy/Buttery

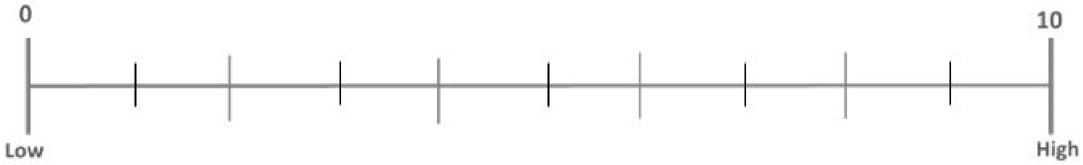

Fruity

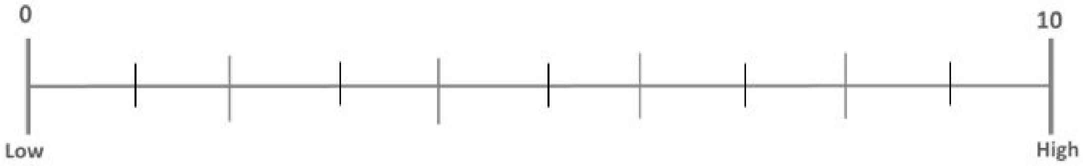

Vegetable

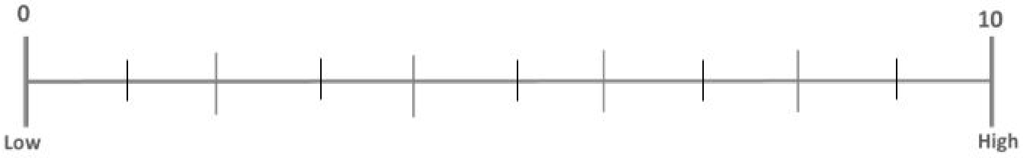

Aftertaste

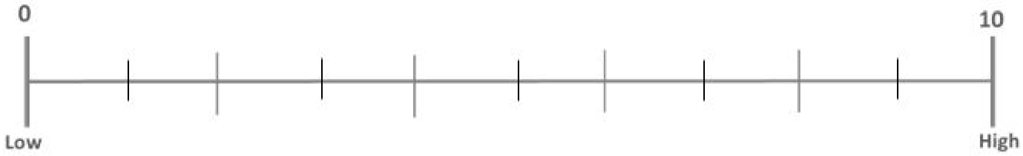

Comments:

____

____

____

Texture

Hardness

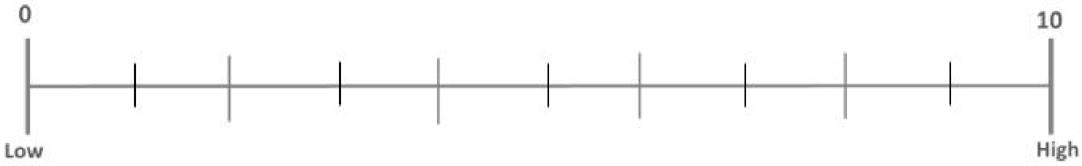

Chewiness

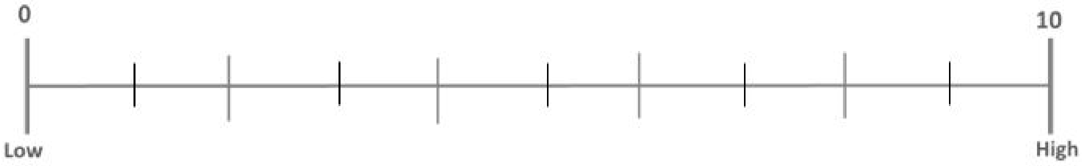

Overall Acceptibility

Acceptibility

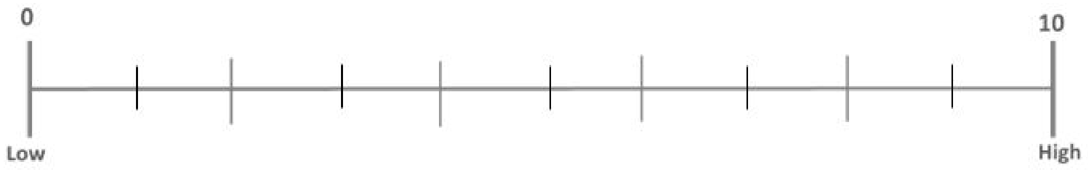

Figure S1. Questionnaire used for sensory analysis.
